# 1097. A Prospective Evaluation of *SARS-CoV-2* Shedding and Evolution in Immunocompromised Hosts During the Omicron Period — IVY Network, 5 U.S. States, April 11, 2022 – February 1, 2023

**DOI:** 10.1093/ofid/ofad500.070

**Published:** 2023-11-27

**Authors:** Zoe M Raglow, Diya Surie, James Chappell, Yuwei Zhu, Emily T Martin, Jennie H Kwon, Anne Frosch, Amira Mohamed, Julie Gilbert, Emily E Bendall, Auden Bahr, Natasha B Halasa, H Keipp Talbot, Carlos G Grijalva, Adrienne Baughman, Kelsey N Womack, Cassandra Johnson, Sydney A Swan, Ashley Burroughs, Emilia Koumans, Meredith L McMorrow, Jennifer L Harcourt, Lydia J Atherton, Natalie J Thornburg, Wesley Self, Adam S Lauring

**Affiliations:** University of Michigan, Ann Arbor, MI; Centers for Disease Control and Prevention, Atlanta, Georgia; Vanderbilt University Medical Center, Nashville, Tennessee; Vanderbilt University, Nashville, Tennessee; University of Michigan, Ann Arbor, MI; Washington University - School of Medicine, St. Louis, MO; Hennepin County Medical Center, Minneapolis, Minnesota; Montefiore medical center, Bronx, New York; University of Michigan, Ann Arbor, MI; University of Michigan, Ann Arbor, MI; University of Michigan Medical School, Ann Arbor, Michigan; Vanderbilt University Medical Center, Nashville, Tennessee; Vanderbilt University Medical Center, Nashville, Tennessee; Vanderbilt University Medical Center, Nashville, Tennessee; Vanderbilt University Medical Center, Nashville, Tennessee; Vanderbilt University Medical Center, Nashville, Tennessee; Vanderbilt University Medical Center, Nashville, Tennessee; Vanderbilt University Medical Center, Nashville, Tennessee; Centers for Disease Control, Atlanta, Georgia; CDC, Atlanta, Georgia; CDC/NCIRD/CORVD/SPB, Atlanta, GA; Division of Viral Diseases, Centers for Disease Control and Prevention (CDC), Atlanta, Georgia; Centers for Disease Control, Atlanta, Georgia; Centers for Disease Control and Prevention, Atlanta, Georgia; Vanderbilt University Medical Center, Nashville, Tennessee; University of Michigan, Ann Arbor, MI

## Abstract

**Background:**

Prolonged SARS-CoV-2 infections in immunocompromised hosts may predict or source the emergence of highly mutated variants of concern. The types of immunosuppression placing patients at highest risk for prolonged infection and associated intra-host viral evolution remain unclear.

**Table 1.**
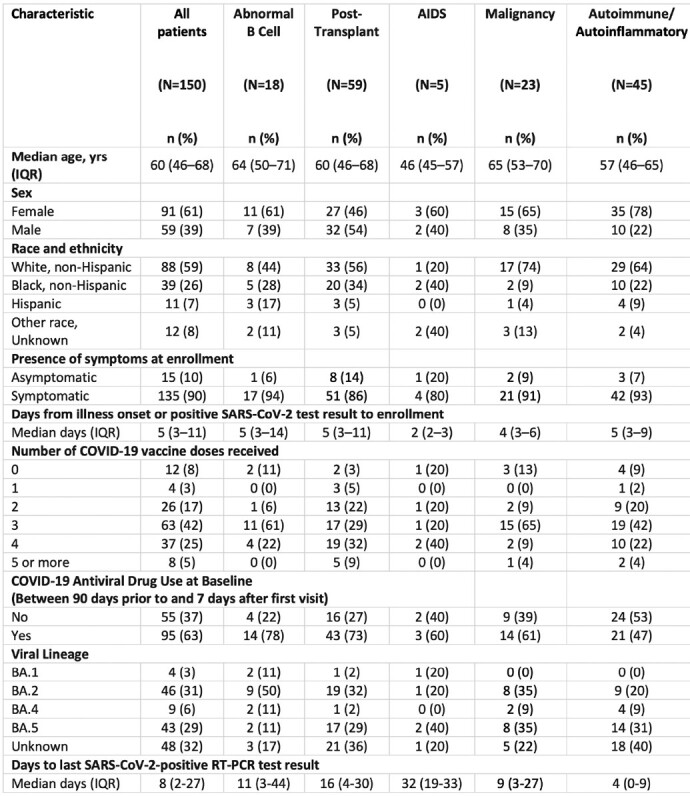
Characteristics at enrollment of immunocompromised patients with SARS-CoV-2 infection — IVY Network, 5 U.S. States, April 11, 2022 – February 1, 2023 Abbreviations: AIDS = acquired immunodeficiency syndrome; IQR = interquartile range; RT-PCR = reverse transcription-polymerase chain reaction

**Figure 1.**
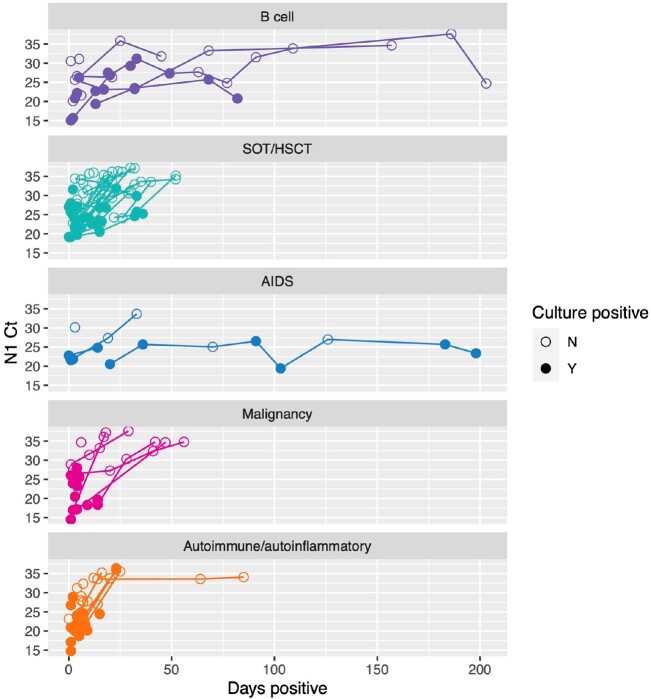
Cycle threshold (Ct) values for SARS-CoV-2 N1 (nucleocapsid) and virus culture isolation over time by immunocompromised group — IVY Network, 5 U.S. States, April 11, 2022 – February 1, 2023, N=150

**Methods:**

Adults aged ≥18 years were enrolled and followed at 5 hospitals in the Investigating Respiratory Viruses in the Acutely Ill (IVY) Network from 4/11/2022 – 2/1/2023. Eligible patients were SARS-CoV-2-positive by RT-qPCR in the previous 14 days and had an immunocompromising condition, including malignancy, solid organ or hematopoietic stem cell transplant (SOT/HSCT), autoimmune/autoinflammatory condition on immunosuppression, AIDS, or primary immunodeficiency. Nasal specimens were collected and tested by RT-qPCR every 2–4 weeks until negative in 2 consecutive specimens. All specimens underwent viral culture and whole genome sequencing. A Cox proportional hazards model was used to assess factors associated with prolonged infection.

**Figure 2.**
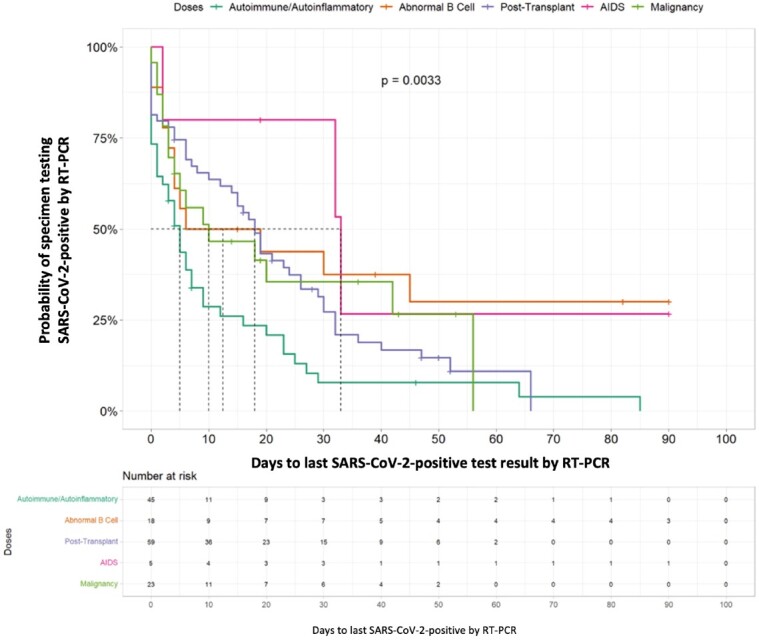
Cox proportional hazards model* depicting association between type of immunosuppression and prolonged SARS-CoV-2 infection by reverse transcription-polymerase chain reaction (RT-PCR) — IVY Network, 5 U.S. States, April 11, 2022 – February 1, 2023, N=150 * Cox proportional hazards model was adjusted for age, sex, race, ethnicity, vaccination history (receipt of any vaccine doses vs. none) and COVID-19 antiviral drug use (receipt of any drugs 90 days before enrollment or 7 days after visit)

**Results:**

150 patients were enrolled with the following conditions: B cell malignancy or anti-B cell therapy (n=18), SOT/HSCT (n=59), AIDS (n=5), non-B cell malignancy (n=23), and autoimmune/autoinflammatory (n=45, Table 1). 37 (25%) were RT-qPCR-positive and 11 (7%) were culture-positive ≥21 days after infection onset. Patients with B cell dysfunction had prolonged infection compared to those with autoimmune/autoinflammatory conditions (aHR 0.28, 95% CI 0.14–0.58) (Figures 1, 2). The within-host evolutionary rate was similar in prolonged (≥ 21 days) and shorter (< 21 days) infections (Figure 3). Consensus spike mutations were identified in 4 individuals who were RT-qPCR-positive ≥ 56 days; 68% were in the receptor-binding domain (RBD) (Figures 4, 5). The common spike mutations in this analysis were rare (< 2%) in global circulation.

**Figure 3A.**
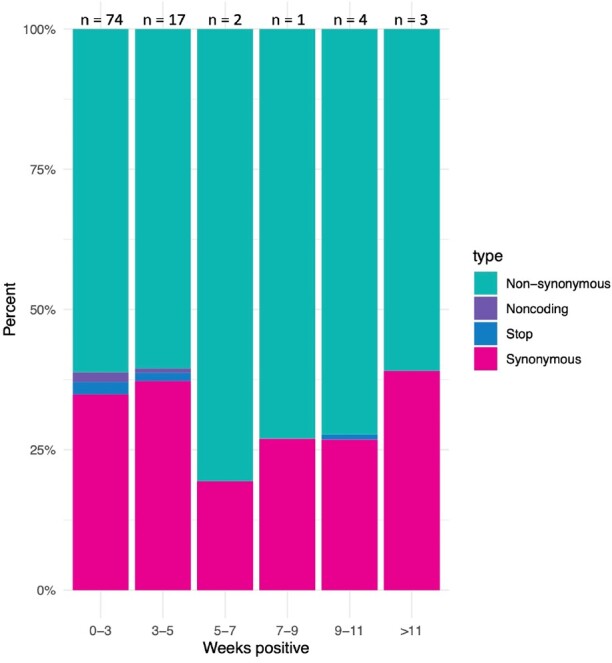
Type of de novo mutations over time among immunocompromised patients with SARS-CoV-2 infection — IVY Network, 5 U.S. States, April 11, 2022 – February 1, 2023, N=150

**Figure 3B.**
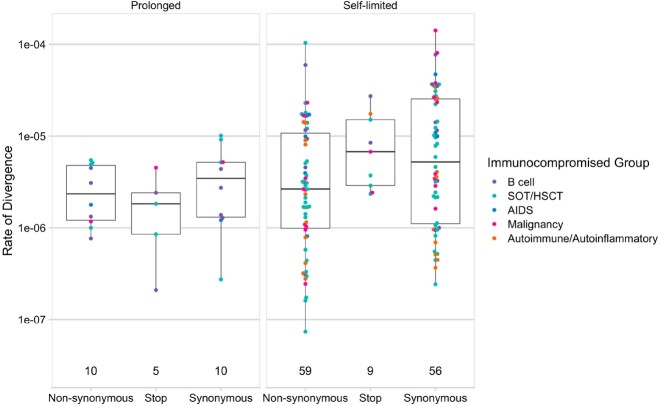
Evolutionary divergence by mutation type in patients with self-limited (<21 days, n = 89) and prolonged (>=21 days, n = 15) SARS-CoV-2 infections — IVY Network, 5 U.S. States, April 11, 2022 – February 1, 2023, N=150

**Figure 4.**
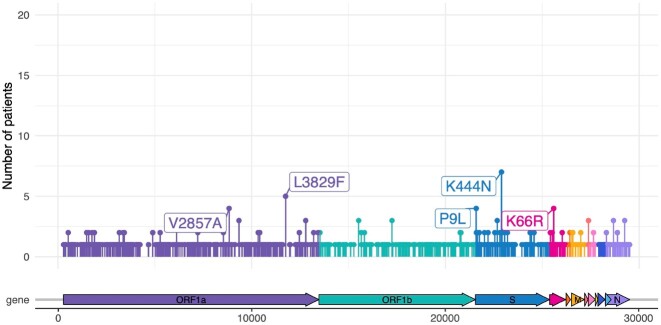
Shared de novo mutations across the entire SARS-CoV-2 genome among immunocompromised patients — IVY Network, 5 U.S. States, April 11, 2022 – February 1, 2023, N=150

**Conclusion:**

In this prospective cohort of immunocompromised patients during the Omicron variant period, prolonged SARS-CoV-2 infections were uncommon. While the within-host evolutionary rates are similar between prolonged and shorter infections, individuals with infections lasting ≥ 56 days accumulated mutations in the spike protein. These appear distinct from those seen globally.

**Figure 5.**
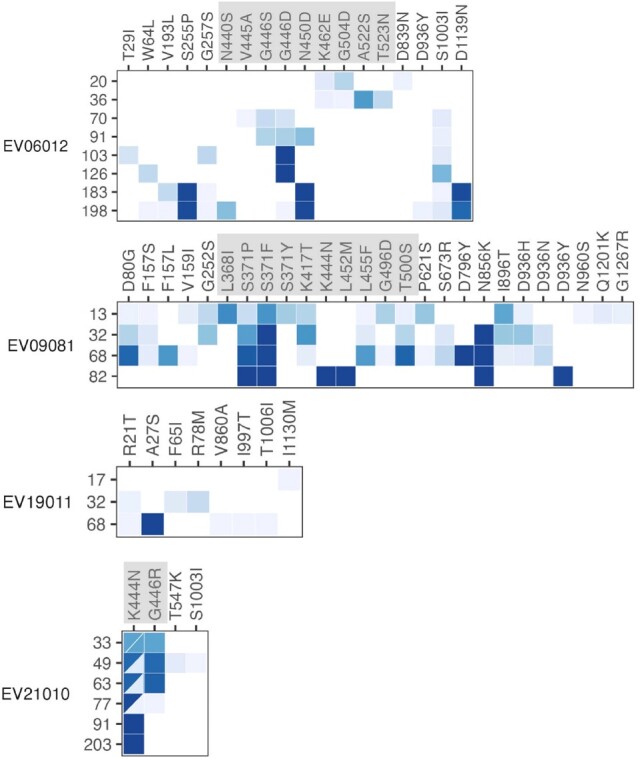
Heatmaps of mutations in SARS-CoV-2 spike protein among immunocompromised patients with ≥2 sequenced specimens and ≥56 days of positive specimens by reverse transcription-polymerase chain reaction (RT-PCR) — IVY Network, 5 U.S. States, April 11, 2022 – February 1, 2023, N=4.

Bisected squares indicate more than one genomic mutation produced the same amino acid mutation. RBD is highlighted in gray.

**Disclosures:**

**Emily T. Martin, PhD, MPH**, Merck: Grant/Research Support **Natasha B. Halasa, MD, MPH**, Merck: Grant/Research Support|Quidell: Grant/Research Support|Quidell: donation of kits|Sanofi: Grant/Research Support|Sanofi: vaccine support **Carlos G. Grijalva, MD, MPH**, AHRQ: Grant/Research Support|CDC: Grant/Research Support|FDA: Grant/Research Support|Merck: Advisor/Consultant|NIH: Grant/Research Support|Syneos Health: Grant/Research Support **Adam S. Lauring, MD, PhD**, Roche: Advisor/Consultant|Sanofi: Advisor/Consultant

